# Who Are You? Views of Self as Reported by 90 Depressed Adolescent Psychiatric Inpatients

**DOI:** 10.31083/AP38761

**Published:** 2025-02-28

**Authors:** Christopher McGovern, James C. Overholser, Christiana Silva

**Affiliations:** ^1^Department of Psychological Sciences, Case Western Reserve University, Cleveland, OH 44106, USA

**Keywords:** self-esteem, depression, suicide

## Abstract

**Background:**

The factors contributing to adolescents’ views of self can be complex and idiosyncratic. Self-esteem can hinge upon a narrow or broad set of factors, depending on how the adolescent conceptualizes the self. The present study examines how narrow and broad views of self may be differentially related to measures of depression severity and suicide risk among adolescents.

**Methods:**

In total, 90 adolescent psychiatric inpatients were evaluated while hospitalized during a major depressive episode. All patients completed the Children’s Depression Inventory, the Hopelessness Scale for Children, the Rosenberg Self-Esteem Scale, and the Self-Esteem Worksheet—an idiographic measure that allows each person to rate the importance and success related to enter their personal values and priorities.

**Results:**

Compared to depressed adolescent inpatients, depressed and suicidal teens reported significantly higher levels of depression and hopelessness, along with significantly lower levels of self-esteem on both measures of self-esteem. Further, lower scores on the Self-Esteem Worksheet were associated with more severe depression, elevated hopelessness, and elevated suicidal ideation.

**Conclusions:**

The Self-Esteem Worksheet provides insights into the mind of vulnerable teens that may help to guide treatment and prevention efforts.

## Main Points

1. Self-esteem can be measured in an idiographic and personalized manner.

2. Low self-esteem was related to more severe depression and elevated hopelessness.

3. Depressed suicidal teens reported somewhat lower self-esteem as compared to their depressed nonsuicidal peers.

## 1. Introduction

A negative view of self is often an important factor in the onset or maintenance of depression. A negative view of self, whether accurate or distorted, is commonly reported by depressed teens and adults. Low levels of self-esteem have been found to be related to higher levels of depression [1], nonsuicidal self-injury [2], suicidal ideation [3] and suicide attempts [4].

Adolescence is an important period for the formation of an individual’s view of self with self-esteem becoming more stable in adulthood [5]. Many factors can play a role in the development of a person’s self-esteem, and a wide range of personal and interpersonal events become important during adolescence. Identifying low self-esteem and intervening early in life is crucial for reducing depression and suicide risk.

Unfortunately, the measurement of self-esteem requires more individualized details than seen with the established assessment tools. Commonly used measures of self-esteem like the Rosenberg Self-Esteem Scale [6], provide estimates of high versus low self-esteem, while omitting any specific elements that may contribute to the person’s view of self. Qualitatively richer measures are needed to help clinicians identify specific intervention points for addressing adolescents’ low self-esteem.

The present study aims to address this gap in self-esteem assessment and intervention in two ways. First, the study aims to examine adolescent views of self as related to measures of depression severity and suicide risk, using an idiographic measure of self-esteem. Second, having a narrow self-view has been postulated to increase risk for extreme fluctuations in self-esteem, thus potentially increasing risk for experiencing depression and suicidality [7]. Therefore, the present study also aims to use the Self-esteem worksheet to examine group differences in depression and suicidality between adolescents with narrow and broad views of self.

## 2. Methods

### 2.1 Participants

The present study evaluated 90 adolescent psychiatric inpatients (46 males, 44 females) during the first week of their hospitalization at a private psychiatric hospital during a major depressive episode. These adolescents ranged in age from 12 to 17, with a Mean age of 15.29 (standard deviation (SD) = 1.26). The vast majority (87.8%) were White, with several participants Black (6.7%) or other racial backgrounds (3.3%). Participants were eligible for the study if they were diagnosed with a major depressive disorder by their attending psychiatrist. The depression was severe enough to require short term hospitalization, while some (n = 40) were also suicidal whereas others (n = 29) did not report suicidal ideation at the time they were admitted to the hospital. Patients were excluded from participation if they had been diagnosed with a psychotic disorder, bipolar disorder, or primary drug abuse. Thus, at the time of the study, all participants were currently diagnosed with a major depressive disorder as recorded by their attending psychiatrist. Most (75%) also reported problems with anxiety, and many (51%) reported ongoing problems with substance abuse. Informed consent was obtained from the patient’s parents or legal guardian, and assent was obtained from the participating adolescent. Participants were assessed within five days of their admission into the hospital.

### 2.2 Central Measure

Self-Esteem Worksheet (SEW) is a one-page idiographic measure of self-esteem completed in collaboration with an interviewer [8]. Participants were provided with instructions for completing the worksheet, following three distinct steps: First, they were asked to list 4–8 major areas of their lives that they felt were related to their view of self. Second, they were asked to rate the subjective importance of each area. These subjective importance ratings are required to sum to a total of 100 across all areas identified. Third, they were asked to rate the self-perceived success in each area. Each self-perceived success rating could range from 0 to 100. After submitting their answers, the Self-Esteem Worksheet component scores were calculated by multiplying the importance rating of each individual area by its respective success rating. After the worksheet was completed, total scores were calculated by summing all component scores and dividing by 100. Self-Esteem Worksheet total scores can range from 0 to 100, with higher scores reflecting a more positive view of self. Based on prior research [8], the Self-Esteem Worksheet has been shown to possess adequate test-retest reliability over a 10-week interval (r[240] = 0.61). In addition, the Self-Esteem Worksheet has also shown concurrent validity. Participants high in self-esteem on the Self-Esteem Worksheet consistently reported lower levels of depression, loneliness, and self-criticism [8].

### 2.3 Additional Measures

Children’s Depression Rating Scale-Revised (CDRS-R) is a 17-item scale used to assess depression severity in adolescents [9]. The CDRS-R is a structured clinical interview that assesses the common symptoms of a major depressive disorder [10]. In the present study, the CDRS-R was used to classify patients as suicidal if they reported both a prior suicide attempt and the presence of suicidal ideation at the time of admission into the hospital. Patients were classified as nonsuicidal if they denied any prior suicidal behavior and the did not report suicidal ideation at the time of admission.

Children’s Depression Inventory (CDI) includes 27 items designed to assess the severity of depressive symptoms [11]. The CDI relies on the patient’s self-report of the most common symptoms of depression as experienced by children and adolescents. Previous studies have shown reliability and validity amongst clinical and non-clinical adolescent populations in various countries [12].

Hopelessness Scale for Children (HSC) includes 17 items that assess hopelessness and expectations toward the future. The HSC evaluates tendencies for pessimism that often underlie suicidal urges [13]. Higher levels of hopelessness have been found in depressed patients with suicidal ideation as compared to depressed patients without suicidal urges [14]. Prior research has shown this scale to be reliable and valid amongst adolescents [15].

Rutgers Alcohol Problem Index (RAPI) assesses 23 areas of negative consequences that may result from one’s alcohol use. Higher scores reflect more problems with alcohol or drug misuse [16]. This scale was originally intended for college students, but has been shown to be both reliable and valid among adolescent [17].

Rosenberg Self-Esteem Scale (RSE) includes 10 items designed to measure self-esteem and how it relates to mental health [6]. This scale has been shown to be reliable and valid amongst adolescents, with no significant differences when compared to the results of adults [18]. Negative views of self on the Rosenberg Self-Esteem Scale have been related to depression severity in adolescent psychiatric patients [19] as well as adult psychiatric patients [20].

## 3. Results

Correlations among the various measures were examined across the entire sample of depressed adolescents (see Table 1). Power analysis suggested that with Alpha set at *p* < 0.05 and a medium effect size, 84 subjects are needed to attain power of 0.80 for correlations. Lower self-esteem (as measured by the Self-Esteem Worksheet) was associated with higher levels of depression (r = –0.39, *p* < 0.001), hopelessness (r = –0.43, *p* < 0.001), and the presence of suicidal thoughts (r = –0.25, *p* < 0.02). Further, scores on the Self-Esteem Worksheet were significantly associated with the Rosenberg Self-Esteem Scale (r = 0.51, *p* < 0.001). There was not a significant association between self-esteem and tendencies for alcohol or drug abuse (r = –0.07, ns).

**Table 1.** **Means, standard deviation and correlations between self-esteem worksheet and measures of distress**.

	*n*	*M*	*SD*	1	2	3	4	5	6
1. Self-Esteem Worksheet	90	68.39	21.92	-					
2. Children’s Depression Inventory	90	12.46	9.62	–0.39***	-				
3. Rosenberg Self-esteem Scale	90	30.46	6.51	0.51***	–0.78***	-			
4. Hopelessness Scale for Children	90	3.81	4.18	–0.43***	0.79***	–0.73***	-		
5. Rutgers Alcohol Problem Index	89	9.04	15.90	–0.07	0.24*	–0.16	0.16	-	
6. Suicidal Ideation	89	0.44	0.50	–0.24***	0.40***	–0.52***	0.55***	–0.02	-

Note: *n* = sample size, *M* = mean, *SD* = standard deviation. ***p < 0.001; *p < 0.05.

The sample was classified into 29 depressed and suicidal patients as compared to 40 depressed suicidal adolescents (see Table 2). Power analysis determined that samples of 30 in each group is acceptable when a larger effect size is expected. Results revealed that suicidal adolescents reported significantly higher levels of depression (t = 4.73, *p* < 0.001), and elevated hopelessness scores (t = 4.24, *p* < 0.001) as compared to the depressed, nonsuicidal patients. Lower levels of self-esteem were found on the Rosenberg Self-Esteem Scale (t = 5.14, *p* < 0.001) and the Self-Esteem Worksheet (t = 1.89, *p* = 0.06) for the suicidal patients compared to the depressed nonsuicidal patients.

**Table 2.** ***t*-test results comparing suicidal and nonsuicidal adolescents**.

Variable	Nonsuicidal		Suicidal		*t*	*p*	Cohen’s *d*
	*M*	*SD*	*M*	*SD*			
Children’s Depression Inventory	6.48	6.02	15.80	10.25	4.38	<0.001***	1.067
Rosenberg Self-Esteem Scale	34.28	3.18	27.78	7.08	4.61	<0.001***	1.125
Hopelessness Scale for Children	1.79	1.82	5.40	4.94	3.74	<0.001***	0.913
Rutgers Alcohol Problem Index ^a^	10.14	13.82	5.55	12.95	1.40	0.17	0.345
Self-Esteem Worksheet	75.27	25.31	66.14	24.67	1.76	0.08	0.429

Note: Sample size across analyses for Suicidal depressed patients (n = 29) and Nonsuicidal depressed patients (n = 40). ^a^ Sample size for Suicidal group (n = 28) due to missing data for one participant. *M* = mean, *SD* = standard deviation. ***p < 0.001.

Additional analyses were conducted to examine group differences between adolescents classified as having a narrow and restricted view of self as compared to adolescents classified as having a broad and diversified view of self (see Table 3). These post hoc analyses relied on small groups, thereby lacking sufficient power. Adolescents were classified as belonging to the narrow view of self group if they reported that one domain on their Self-esteem Worksheet captured at least 75% of their view of self. Adolescents were classified as belonging to the broad view of self group if they had reported no domain on their Self-Esteem Worksheet reflected more than 40% of their view of self. Independent samples *t*-test and Chi-square analyses revealed no significant differences between the narrow and broad self-view groups on their self-reported levels of depression, self-esteem, hopelessness, presence of prior suicide attempt, and presence of suicide ideation at the time of the study.

**Table 3.** ***t*-test results comparing adolescents with broad and narrow views of self**.

Variable	Broad		Narrow		*t*	*p*	Cohen’s *d*
	*M*	*SD*	*M*	*SD*			
Children’s Depression Inventory	11.45	6.94	14.52	12.31	0.98	0.33	0.306
Rosenberg Self-Esteem Scale	31.20	5.07	28.57	7.96	1.25	0.22	0.392
Hopelessness Scale for Children	3.35	2.91	4.62	4.89	1.00	0.32	0.313

Note: Sample size across analyses for Broad view of self (*n* = 20) and Narrow view of self (*n* = 21) groups. *M* = mean, *SD* = standard deviation.

## 4. Discussion

Low self-esteem can play a role in promoting depression and suicide risk among teens. The present findings suggest that low self-esteem is associated with greater depression severity, hopelessness and the presence of suicide ideation. In addition, the present findings indicate that as compared to depressed nonsuicidal teens, depressed suicidal teens report lower self-esteem, as well as higher levels of both depression severity and hopelessness. Notably these findings remained significant when self-esteem was classified based on the idiographic measure of self-esteem using the Self-Esteem Worksheet. Furthermore, the Self-Esteem Worksheet was highly correlated with the well-established Rosenberg Self-Esteem Scale, while also providing a richer picture of a person’s view of self as compared the information obtained through structured questionnaires. Interestingly, teens with a narrow view of self did not differ from teens with a broad self-view on measures of depression severity, self-esteem, hopelessness and history of suicidality. However, the small sample size used to examine groups differences may limit the interpretability of the results.

## 5. Conclusions

Overall, the present findings highlight the role of self-esteem in depression and suicide risk. Depression and hopelessness serve as important proximal risk factors that reflect the acute distress often associated with a suicidal crisis. However, low self-esteem may provide an underlying risk factor that could remain present over long periods of time [5]. Traditional clinical tools such as the Rosenberg Self-esteem scale, while effective at measuring adolescent’s self-esteem, fail to provide insight into the life factors responsible for the adolescent’s current self-conception. Being an idiographic measure, the Self-Esteem Worksheet provides clinicians with valuable information regarding individualized sources of self-esteem, which can act as specific treatment targets for those adolescents’ struggling to form of a healthy view of self.

## Availability of Data and Materials

Research materials are available upon request. Data files are stored in a secure setting. Because of HIPAA law protecting electronic information about patients, the original data cannot be shared publicly.
